# Extraordinary Case of Opium Eating Cured

**Published:** 1855-03

**Authors:** W. H. Myers

**Affiliations:** Loudonville, Ashland Co., Ohio


*Extraordinary case of Opium Eating Cured.* By W. H. MYERS, of Loudonville, Ashland Co., Ohio.

In the habitual use of the narcotic opium, nothing is more striking than the facility with which the system bears its gradual increase, each successive dose being less efficient than the preceding one, if delayed until the action of the former has terminated. The truth of this will be made sufficiently evident by the following case:

In the fall of 1853, a physician came under my observation who had habitually used opium as a stimulus for *six years*. Its effects during the first part of this period were, as usually described, exhilarating; a calm, cheerful, hopeful state of feeling existing, with freedom from care, dreamy reverie, and pleasing hallucinations. But these had long ceased, and he was now suffering pangs from the same drug that had afforded him such varied enjoyment, all its pleasures being replaced by morbid fancies and sensations of the most unpleasant character. At this time he was using the enormous quantity of from *fifteen to eighteen* grains of the sulphate of morphia in twenty-four hours, taken at intervals of four hours and continued during the night. Thus, the repetition of the stimulus followed so quickly and regularly, as to anticipate the appearance of the consecutive debility, the unavoidable consequence of its abstraction.

I will only describe a few of the most prominent symptoms:—one of these was a constant and fixed delusion, presaging impending death ; this feeling haunted him day and night, accompanied with a morbid dread, sometimes definite and often ill defined. His behaviour, temper, spirits and conversation partook of this predominant belief, with a disposition to intrude the subject which was the basis of his thoughts. Accompanying this condition was a belief that his intellect was disordered, or becoming so ; loss of control over his mental operations ; neglect of business ; walking restlessly at night ; sleep almost banished ; terrific and distressing dreams ; altered sensibility ; numbness ; pricking in the hands and fingers ; tinnitus aurium ; hallucination of voices and conversations in his presence; muscæ volitantes; phantoms or fancied visions; vertigo; muscular contractions; uncertain walk ; diminished strength ; inability to stand or rise in bed, and varied action of the heart as regards its frequency, and force. The abdominal symptoms were sense of sinking, weight, fulness and dragging, accompanied by unusual constipation, and a distaste for food.

After treatment had commenced, a set of new symptoms were developed ; his bladder, became excessively irritable, accompanied with profuse diarrhœa and gastric disturbance, nausea, vomiting and great prostration; with a disagreeable sense of formication in the scalp. While in this condition attempts to quit the injurious habit were made, but ineffectually. A number of physicians of celebrity were consulted, who all suggested a recourse to substitutes. Extracts of belladonna, aconite and cannabis were tried, but without deriving the least benefit from their use. Reason was already partially dethroned, and death seemed inevitable. After all these repeated failures I addressed a letter to Prof. Dunglison, of Philadelphia, asking his advice, who, in reply, wrote as follows:

*Philadelphia, February 17th,* 1853.

Dear Sir,—There is no safety for your friend, except in a fixed determination to diminish daily the amount of the narcotic, not by guess, but by absolute weighing out of the daily allowance. To give it up all at once, would be followed by most distressing phenomena; but if he gradually diminishes the amount consumed, the privation may be tolerated, and the pernicious evil be ultimately got rid of. It will require, however, the most *dogged* resolution to carry into effect this plan, otherwise it must fail. In the depression and sinking that may arise, he may have recourse, three or four times a day, to from forty to sixty drops of the *spiritus ammoniæ fœtidus*, of the London and Edinburgh Pharmacopœias, formulæ for which are to be found in the Dispensatory of the United States. The further details of treatment will suggest themselves to you. The most important thing is to establish a clear indication, and to let nothing interfere with its being energetically fulfilled. No substitute can be used for the narcotic which will not be ultimately as injurious; and experience has taught me that the plan I have suggested to you has been entirely satisfactory and successful. That it may be so in the case on which you consult me, is the sincere wish of your obedient and humble servant,

ROBLEY DUNGLISON.

This plan, persevered in with the most determined resolution, diminishing, almost imperceptibly, the dose from day to day, was entirely successful in twenty weeks in reclaiming our victim, and restoring him to the society of his friends in full possession of his faculties and in the enjoyment of good health, entirely cured of the habit of opium eating, and free from all its evil effects.

